# Covalent Cysteine Targeting of Bruton’s Tyrosine Kinase (BTK) Family by Withaferin-A Reduces Survival of Glucocorticoid-Resistant Multiple Myeloma MM1 Cells

**DOI:** 10.3390/cancers13071618

**Published:** 2021-03-31

**Authors:** Emilie Logie, Chandra S. Chirumamilla, Claudina Perez-Novo, Priyanka Shaw, Ken Declerck, Ajay Palagani, Savithri Rangarajan, Bart Cuypers, Nicolas De Neuter, Fazil Mobashar Hussain Urf Turabe, Navin Kumar Verma, Annemie Bogaerts, Kris Laukens, Fritz Offner, Pieter Van Vlierberghe, Xaveer Van Ostade, Wim Vanden Berghe

**Affiliations:** 1 Laboratory of Protein Science, Proteomics and Epigenetic Signaling (PPES) and Integrated Personalized and Precision Oncology Network (IPPON), Department of Biomedical Sciences, University of Antwerp, Campus Drie Eiken, Universiteitsplein 1, 2610 Wilrijk, Belgium; chandra.ace@gmail.com (C.S.C.); claudina.pereznovo@uantwerpen.be (C.P.-N.); kendeclerck90@hotmail.com (K.D.); ajaypalagani@gmail.com (A.P.); xaveer.vanostade@uantwerpen.be (X.V.O.); 2 Plasma Lab for Applications in Sustainability and Medicine Antwerp (PLASMANT), Department of Chemistry, University of Antwerp, 2610 Wilrijk, Belgium; priyanka.shaw@uantwerpen.be (P.S.); annemie.bogaerts@uantwerpen.be (A.B.); 3 PamGene International B.V., 5211 Hertogenbosch, The Netherlands; srangarajan@pamgene.com; 4 Biomedical Informatics Network Antwerp (Biomina), Department of Informatics, University of Antwerp, 2610 Wilrijk, Belgium; bart.cuypers@uantwerpen.be (B.C.); nicolas.deneuter@uantwerpen.be (N.D.N.); kris.laukens@uantwerpen.be (K.L.); 5 Lymphocyte Signaling Research Laboratory, Lee Kong Chian School of Medicine, Nanyang Technological University Singapore, Singapore 1308232, Singapore; fazil.turabe@gmail.com (F.M.H.U.T.); nkverma@ntu.edu.sg (N.K.V.); 6 Hematology, Department of Internal Medicine, Ghent University, 9000 Ghent, Belgium; fritz.offner@ugent.be; 7 Department of Biomolecular Medicine, Ghent University, 9000 Ghent, Belgium; pieter.vanvlierberghe@ugent.be

**Keywords:** withaferin A, BTK, multiple myeloma, therapy resistance, glucocorticoids, ibrutinib

## Abstract

**Simple Summary:**

Glucocorticoid therapy resistance in B-cell malignancies is often associated with constitutive activation of tyrosine kinases. Novel anticancer drugs targeting hyperactivated tyrosine kinases, such as Bruton’s tyrosine kinase (BTK), have, therefore, gained much interest over the past few decades and have already been approved for clinical use. In this study, we compared the therapeutic efficacy of the phytochemical kinase inhibitor withaferin A with the clinically approved BTK inhibitor ibrutinib to target hyperactivated tyrosine kinase signaling in glucocorticoid-resistant multiple myeloma cells. Our results demonstrate that withaferin A-induced cell death of glucocorticoid-resistant MM1R cells involves covalent cysteine targeting of multiple Hinge-6 domain type tyrosine kinases of the kinase cysteinome classification, including BTK.

**Abstract:**

Multiple myeloma (MM) is a hematological malignancy characterized by plasma cells’ uncontrolled growth. The major barrier in treating MM is the occurrence of primary and acquired therapy resistance to anticancer drugs. Often, this therapy resistance is associated with constitutive hyperactivation of tyrosine kinase signaling. Novel covalent kinase inhibitors, such as the clinically approved BTK inhibitor ibrutinib (IBR) and the preclinical phytochemical withaferin A (WA), have, therefore, gained pharmaceutical interest. Remarkably, WA is more effective than IBR in killing BTK-overexpressing glucocorticoid (GC)-resistant MM1R cells. To further characterize the kinase inhibitor profiles of WA and IBR in GC-resistant MM cells, we applied phosphopeptidome- and transcriptome-specific tyrosine kinome profiling. In contrast to IBR, WA was found to reverse BTK overexpression in GC-resistant MM1R cells. Furthermore, WA-induced cell death involves covalent cysteine targeting of Hinge-6 domain type tyrosine kinases of the kinase cysteinome classification, including inhibition of the hyperactivated BTK. Covalent interaction between WA and BTK could further be confirmed by biotin-based affinity purification and confocal microscopy. Similarly, molecular modeling suggests WA preferably targets conserved cysteines in the Hinge-6 region of the kinase cysteinome classification, favoring inhibition of multiple B-cell receptors (BCR) family kinases. Altogether, we show that WA’s promiscuous inhibition of multiple BTK family tyrosine kinases represents a highly effective strategy to overcome GC-therapy resistance in MM.

## 1. Introduction

MM is a hematological malignancy of terminally differentiated plasma cells and is currently the second most common adult blood cancer [1]. MM often results in development of end-organ diseases, such as anemia, hypercalcemia, renal insufficiency, and bone lesions, making it an illness of considerable clinical and social impact [2,3]. In recent years, important therapeutical advancements in the field of MM have been made, increasing the life expectancy of patients by six to ten years [4,5]. These novel therapies have mostly been developed based on an improved understanding of the biology of myeloma cells and their interaction with the bone marrow (BM) environment [6]. They include proteasome inhibitors [7], immunomodulatory drugs [8], glucocorticoids (GCs) [9], monoclonal antibodies [10], and histone deacetylase inhibitors [11]. However, MM still remains an incurable disease as the majority of patients eventually relapse and become refractory to existing therapies [12]. Acquisition of resistance to anticancer drugs, therefore, remains the main barrier in treating MM [4,13].

One of the cellular pathways mediating drug resistance in many B-cell malignancies is the BCR signaling pathway [14]. Under physiological conditions, the BCR is activated upon ligation of antigen and promotes survival, function and development of B-cells [15]. After initial antigen binding, the immune receptor tyrosine activation motif domains CD79A and CD79B are phosphorylated by Src family kinases Lyn and Syk and recruit other adaptor proteins and tyrosine kinases (TK), a key example being Bruton’s tyrosine kinase (BTK) [16]. BTK ultimately orchestrates activation of downstream effectors of BCR signaling, such as nuclear factor-κB (NF-κB) and nuclear factor of activated T cells (NFAT), through PLC-γ2 and phosphoinositide 3-kinase (PI3K) phosphorylation [14]. In MM, BTK is often constitutively activated, thereby modulating survival signals and therapy resistance [17,18,19,20]. As a result, BTK kinase inhibitors have received growing pharmacological interest and have already shown promising therapeutic responses in B-cell malignancies in the clinic [21]. More particularly, the covalent-binding BTK inhibitor IBR has shown to be a potent anticancer drug in chronic lymphocytic leukemia (CLL), mantle cell lymphoma, diffuse large B-cell lymphoma, and MM by interfering with B-cell homing, survival and microenvironment-mediated drug resistance [17,18,22,23,24,25]. Suppression of BTK hyperactivation is also key to the therapeutic efficacy of GCs in B-cell leukemias, where IBR has been shown to improve GC therapy response [26,27,28,29]. In clinical trials of MM, combination therapies investigating IBR efficiency demonstrated encouraging responses and a manageable safety profile [30,31].

Although preliminary clinical data revealed the beneficial effects and acceptable safety profile of IBR in MM, clinical studies in other B-cell malignancies have linked IBR use with adverse effects, including diarrhea, fatigue, nausea, and rashes [32,33]. More important, IBR therapy is associated with a significant increase in the occurrence of ventricular arrhythmias and sudden cardiac death [34,35]. The underlying mechanisms of these severe side effects are not well understood but could be partially explained by the off-targets interaction of IBR with interleukin-2-inducible T-cell kinase (ITK), epidermal growth factor (EGFR), and PI3K [36]. Another pressing issue that has arisen since IBR has been applied in the clinic is the development of therapy resistance (reviewed in [37]). Nearly one-third of patients diagnosed with B-cell malignancies display primary resistance against IBR therapy, while many others acquire resistance over time. These major issues accompanying the therapeutic use of IBR have sparked the development of second and third-generation BTK inhibitors (e.g., acalabrutinib), characterized by higher selectivity and potency profiles [38]. Unfortunately, acquired resistance to these novel BTK inhibitors has already been described in some B-cell malignancies as well [39]. As a result, alternative treatment strategies to suppress BTK hyperactivation in therapy-resistant hematological malignancies are currently being investigated in high-throughput combinatorial screenings of clinically approved and preclinical investigational compound libraries [40]. Given that nearly half of the agents used in cancer therapy today are either natural products or derivatives thereof, novel BTK-targeting lead compounds may be identified from this vast arsenal of chemically active structures [41,42,43]. Interestingly, withaferin A, a withanolide phytochemical isolated from *Withania somnifera,* is one of the top investigational compounds prioritized for IBR combination therapy to target chronic active BCR signaling [40].

WA reveals broad-spectrum therapeutic activities in several (drug-resistant) cancer cell types [44], including B-cell lymphoma and MM [45,46,47]. Of particular interest, some of WA’s antitumor effects have been attributed to its ability to covalently target kinase activity [48,49,50,51,52]. Accordingly, innovative phosphopeptidome kinome activity profiling, RNA sequencing, in silico docking simulations, and chemo-affinity approaches were combined in this study to characterize BTK hyperactivation and TK inhibitor therapy response of WA and IBR in GC-resistant MM cells.

## 2. Results

### 2.1. GC Therapy Resistance in Multiple Myeloma Is Associated with Hyperactivation of Tyrosine Kinases

**Figure 1:**
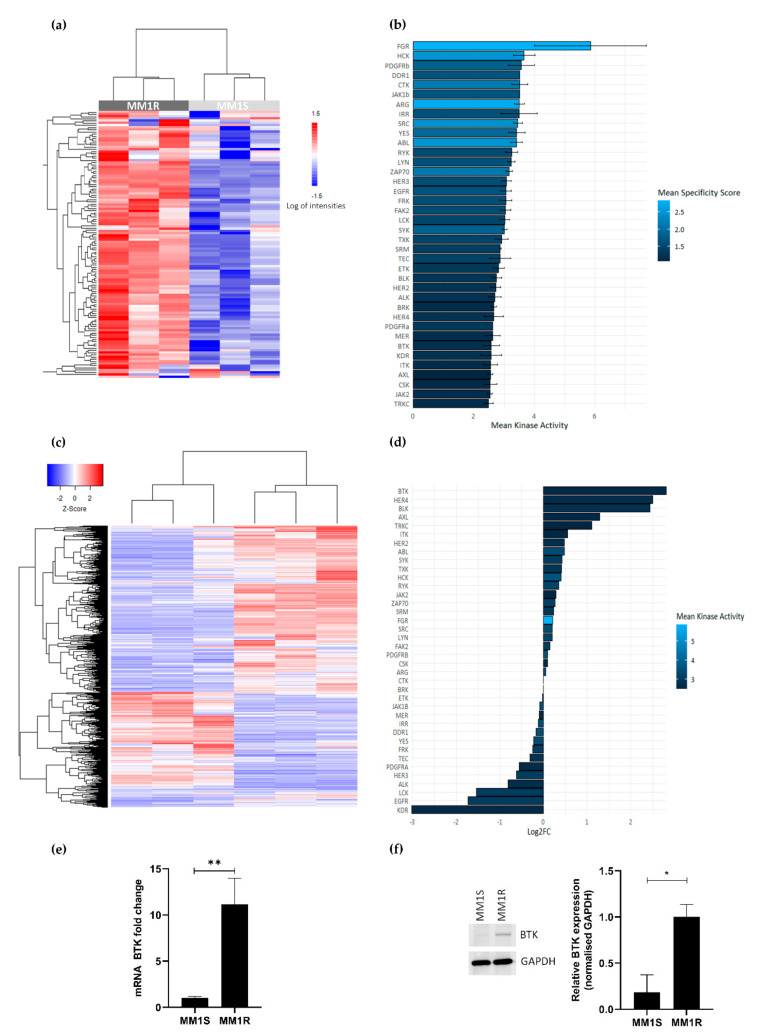
(**a**) Heatmap showing phosphorylation intensities of peptides serving as substrates for tyrosine kinases. Figure shows hyperphosphorylated (red) or hypo-phosphorylated (blue) peptides in MM1R (*n* = 3) and MM1S (*n* = 3) samples. (**b**) Ranking of hyperactivated kinases in MM1R versus MM1S cells based on the top 20 significant differentially phosphorylated peptides. Fill color of the bars is based on the kinase specificity score, indicating the specificity of differences in kinase activity with respect to the quantity of peptides used for predicting the corresponding kinase (**c**) Heatmap representation of differentially expressed genes (logFC >|1|, *p* < 0.01) in MM1R versus MM1S cells as determined by RNA sequencing. *n* = 3 biologically independent replicates per cell line. (**d**) Ranking of the top overexpressed kinases in MM1R versus MM1S cells based on their log2-fold change as determined by RNA sequencing. Fill colors of the bars are a measure for kinase activity as measured via the PTK-specific phosphopeptide array. (**e**) Relative Bruton’s tyrosine kinase (BTK) mRNA levels in MM1R and MM1S cells. Data are plotted as the mean ± s.d., *n* = 3 biologically independent replicates (** *p* = 0.0035, unpaired *t*-test). (**f**) Western immunoblot detection and quantification of basal BTK and GAPDH protein levels in MM1R and MM1S cells. Data are plotted as the mean ± s.d., *n* = 3 biologically independent replicates (* *p* = 0.0385, unpaired *t*-test).

GC therapy-sensitive MM1S and -resistant MM1R cell lines derived from a single MM patient have previously been described as cell models to study the etiology of GC therapy resistance and to evaluate novel classes of chemotherapeutic drugs [53,54]. To investigate the vulnerability of GC-resistant MM1R cells for specific clinical TK inhibitor drugs, we compared the tyrosine kinome activity profiles of GC-resistant MM1R and GC-sensitive MM1S cell lysates by means of a PTK-specific phosphopeptide array (PamChip), containing 144 conserved peptides corresponding to TK specific substrates [55,56]. Overall, TK activity was consistently higher in MM1R cells compared to MM1S cells (Figure 1a and Figure S1). Identification of the 20 most significant differential hyperphosphorylated peptides (adjusted *p*-value (FDR) < 0.01) in MM1R compared to MM1S predicted hyperactivation of multiple (non) receptor TK in MM1R, such as SYK, DDR, ABL, ZAP70, FAK2, BRK, BTK, ITK and FGR (Figure 1b). Subsequent MetaCore pathway analysis showed that the hyperactivated kinases in MM1R cells are involved in cell proliferation, cell cycle regulation, cell adhesion, cancer, therapy resistance, immune response, T-cell receptor signaling and BCR signaling (Supplementary Figure S2).

To investigate whether the observed TK hyperactivation is also reflected at the transcriptome level, RNA sequencing analysis of basal gene expression in MM1S and MM1R cells was conducted. Results were analyzed with the R-package DESeq2 [57], using a selection criterion of a minimal FDR < 0.01. Upon comparing gene expression patterns in both cell lines, 1383 differentially expressed genes (DEG) (logFC > 1 or logFC < −1) could be identified (Figure 1c). Interestingly, from all hyperactivated TK in MM1R cells listed before, BTK was identified as the strongest upregulated TK in GC-resistant MM1R cells (Figure 1d, is included in the top 20 most significant DEG (Table 1). Upregulation of BTK in MM1R cells was validated by qPCR and Western blot analysis (Figure 1e–f) and is in line with previous observations [17,58]. Taken together, these findings suggest that BCR signaling and BTK in MM1R cells may represent an attractive target to kill GC-resistant MM1R cells.

### 2.2. The Tyrosine Kinase Inhibitor Profile of Withaferin A and Ibrutinib Show a High Degree of Similarity

**Figure 2:**
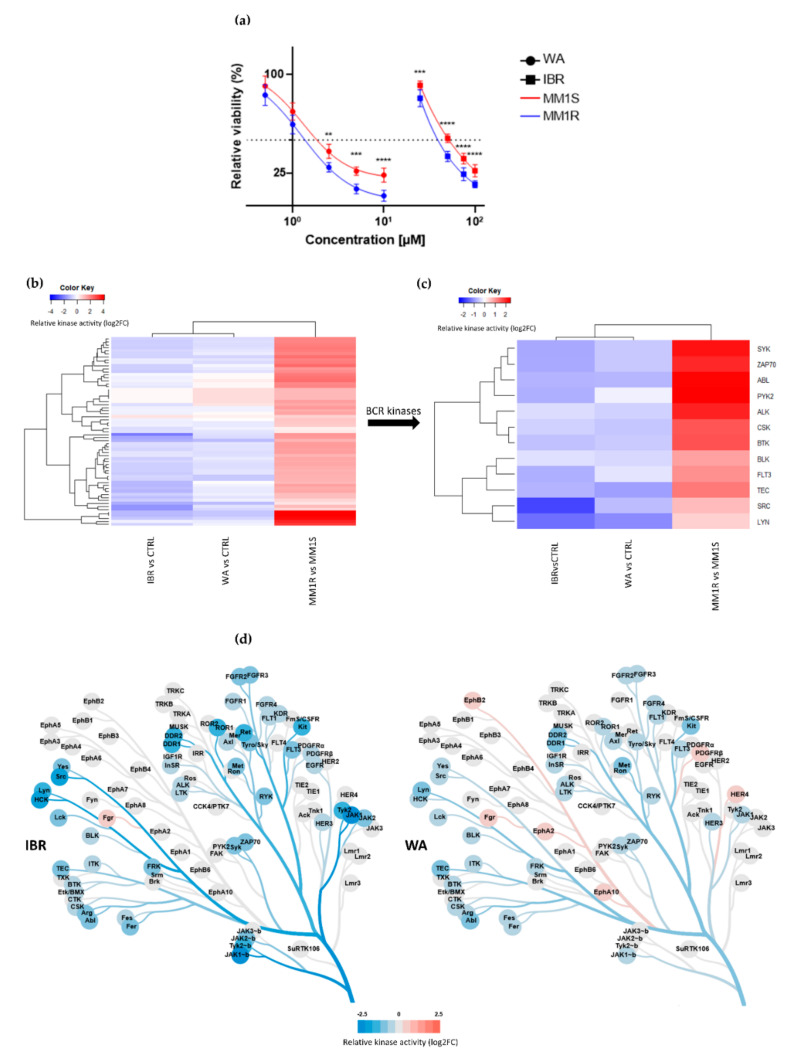
(**a**) Relative cell viability of MM1 cells upon 24 h exposure to increasing concentrations of BTK inhibitor ibrutinib (IBR) or withaferin A (WA). Data are plotted as the mean ± s.d., *n* = 3 biologically independent replicates. (** *p* < 0.01, *** *p* < 0.001 **** *p* < 0.0001, ANOVA). (**b**) Heatmap representation of hyperactivated or inhibited kinases in MM1R versus MM1S cells, or following 15 min IBR or WA treatment, *n* = 3 biologically independent replicates per treatment group. (**c**) Close-up heatmap representation of Figure 2b showing inhibited BCR-related kinases in MM1R versus MM1S or following 15 min IBR or WA treatment, *n* = 3 biologically independent replicates per treatment group. (**d**) Kinase trees displaying the tyrosine kinase targets of IBR (left) and WA (right). Kinase trees were generated with the CORAL web tool (http://phanstiel-lab.med.unc.edu/CORAL/, accessed on 30 November 2020).

Taking into account the hyperactivation of BCR-BTK kinase signaling in MM1R cells (see 2.1.), we next tested their sensitivity for the clinically approved BTK inhibitor IBR as well as WA, a top prioritized investigational phytotherapeutic compound identified in a high-throughput drug screening against chronic BCR signaling [40]. MM1R cells were treated for 24 h with different concentrations of WA or IBR kinase inhibitors, and the relative % cell survival/cell death was evaluated by MTT assay. Both compounds were effective in killing GC-resistant MM1R cells in a dose-dependent manner, although WA is the more potent cell death inducer (IC50 = 1.7 µM) since its IC50 was > 10 times lower than the one of IBR (IC50 = 27.9 µM) (Figure 2a). Remarkably, both WA and IBR also induce cell death in GC-sensitive MM1S cells lacking BTK overexpression, although IC50 values were higher than MM1R cells (IC50_WA_ = 1.9 µM, IC50_IBR_ = 49.3 µM) (Figure 2a).

To explore the mode of action of these compounds, we measured corresponding cellular changes in TK activities by phosphopeptidome based tyrosine kinome profiling of MM1R cells exposed to either WA (1 µM) or IBR (1 µM). Similarly, and as mentioned above, cell lysates of treated cells were analyzed through PTK-specific phospho-peptide arrays, after which the top activated or inhibited kinases were identified based on the significant differences in phospho-intensities of the PTK peptide substrates. We found that WA and IBR both inhibited most of the hyperactivated kinases in MM1R cells, with largely overlapping, though promiscuous, TK inhibitor profiles (Figure 2b, annotation of heatmap rows can be found in Supplementary Figure S3). At the level of BCR signaling, clear inhibition of BTK kinase activity can be observed in the presence of IBR, as expected, and WA (Figure 2c,d). Besides BTK, multiple BCR signaling kinases, such as ZAP70, BLK, FLT3, TEC, and SYK, are also targeted by both WA and IBR (Figure 2c). In line with previous studies, which already revealed that IBR could trigger off-target BCR-BTK independent kinase inhibitor (side) effects [59], we also identified additional IBR-responsive TK in MM1R cells (Figure 2c, Figure S3). Of special note, whereas most binding affinities of IBR have been determined in vitro (Supplementary Figure S4), we provide the first cell-based integrated tyrosine kinome activity map in MM1R cells in the presence of the IBR inhibitor. Although WA inhibits similar BCR family kinases as IBR, variations in kinase inhibitor specificity/potency of WA in comparison to IBR treatment may explain differences in the therapeutic efficacy of both compounds in MM1R cells (Figure 2a,d).

### 2.3. WA Inhibits BCR-BTK Kinase Activity by Transcriptional Downregulation and Covalent Cysteine-Dependent Targeting of BTK

**Figure 3:**
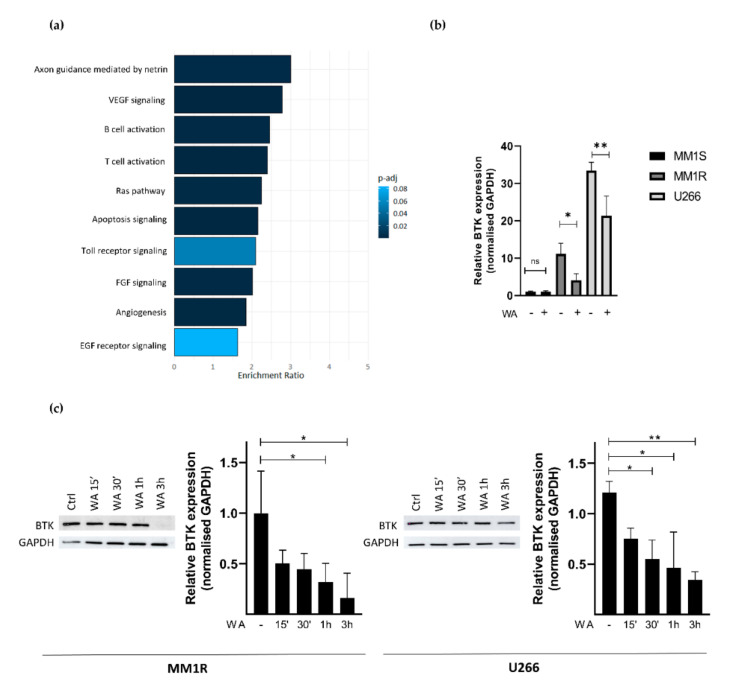
(**a**) Panther pathway enrichment analysis of significant (FDR < 0.05) differentially expressed genes of WA-treated MM1R cells as determined by RNA sequencing. (**b**) Relative BTK mRNA levels of MM1R, MM1S and U266 cells treated with WA for 3 h. Data are plotted as the mean ± s.d., *n* = 3 biologically independent replicates (* *p* = 0.0453, ** *p* = 0.0015, ANOVA) (**c**) Western blot detection and quantification of BTK and GAPDH expression levels after WA treatment in MM1R and U266 cells. Data are plotted as the mean ± s.d., *n* = 3 biologically independent replicates. (* *p* < 0.05, ** *p* < 0.01, ANOVA).

To further characterize how WA decreases BTK kinase activity in MM1R cells, we next checked whether WA treatment changes BTK mRNA and protein expression levels. Panther pathway enrichment analysis of RNA sequencing data from WA-treated MM1R cells already revealed that DEGs are significantly enriched in B-cell activation (Figure 3a). More particularly, WA significantly decreased BTK mRNA expression (Log2FC = −0.525, FDR = 0.023). This was further confirmed by qPCR and western immunoblot experiments. As can be observed from Figure 3b,c, WA was able to lower BTK expression in a time-dependent manner, both at the mRNA and protein level. Similar WA-specific changes could also be observed in U266 cells, another GC-resistant multiple myeloma cell line sensitive to WA treatment (Figure 3b,c and Figure S5). In line with previous studies [60,61,62], we did not observe any changes in BTK expression after the IBR treatment of MM1R cells indicating that IBR mainly targets BTK (hyper)phosphorylation and not total BTK protein levels (Supplementary Figure S6).

**Figure 4:**
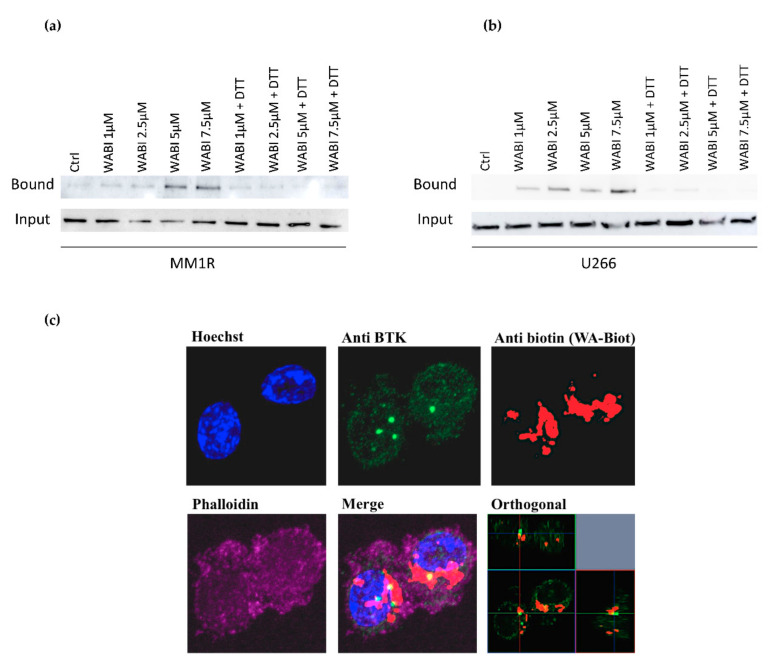
(**a**) Western immunoblot detection of BTK levels before and after pulldown with biotinylated WA (WABI), following 2 h WABI treatment in MM1R in the presence or absence of excess thiol donor DTT (1 mM). (**b**) Western immunoblot detection of BTK levels before and after pulldown with biotinylated WA (WABI), following 2 h WABI treatment in U266 cells in the presence or absence of excess thiol donor DTT (1 mM). (**c**) Confocal imaging of colocalization of BTK expression and WABI localization in MM1R cells.

Since WA contains several reactive nucleophilic groups, which can covalently bind to kinase sulfhydryl groups of cysteines through Michael addition [48,63,64], we also evaluated the potential covalent binding of WA to BTK by pulldown experiments with biotinylated WA (WABI). Pull-down experiments with WABI in MM1R cells indeed confirmed cysteine-dependent binding to BTK, which can be blocked by excess amounts (1 mM) of the reducing agent dithiothreitol (DTT) (Figure 4a). Along the same line, covalent WA-BTK interaction could be confirmed in U266 cells (Figure 4b). The biological relevance of this covalent interaction could further be validated in washout experiments where MM1R cells were exposed to increasing concentrations of WA for 15 min, after which it was washed away with PBS. Although WA cells were only briefly treated with WA, the cell viability of MM1R cells was still affected in a dose-dependent manner 24 h post-treatment (Supplementary Figure S7). The growth inhibition remains the strongest in the unwashed cells, suggesting the contribution of non-covalent interactions of WA as well. H-bond and van der Waals interactions between WA and target molecules, such as Hsp90, have indeed been reported to contribute to the anticancer mechanism of WA [65]. Alternatively, covalent binding by WA may be weaker than a normal covalent binding and become reversed by washout experiments [66,67,68]. Finally, by confocal microscopy, we could also demonstrate colocalization of BTK and WABI in MM1R cells (Figure 4c).

### 2.4. Covalent C481 Targeting of BTK by WA in Hinge-6 Domain of the Protein Kinase Cysteinome Classification Reduces Survival of Glucocorticoid Resistant Multiple Myeloma MM1 Cells

**Figure 5:**
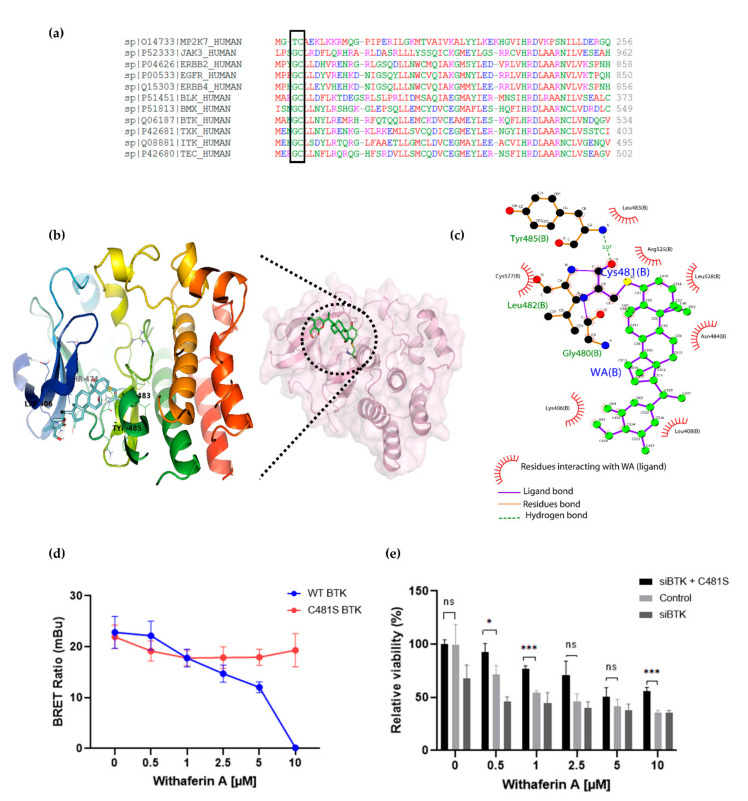
(**a**) Multiple sequence alignment of Hinge 6 domain-type kinases. The conserved GC-motif between different orthologs is indicated. Alignment was performed with Clustal Omega (https://www.ebi.ac.uk/Tools/msa/clustalo/ accessed on 30 November 2020). (**b**) Crystal structure of BTK (PBD id: 6TFP) in complex with WA (PubChem CID: 26537) covalently bound to Cys481. (**c**) Interaction between WA and BTK structure visualized using Ligplot [74], showing the covalent bond formation of the C4-OH group of WA with the SH group of Cys481 from BTK. (**d**) Inhibition of wild-type (WT) BTK and mutated (C481S) BTK by WA in HEK-293 cells. Data are plotted as mean ± s.d., *n* = 3 biologically independent replicates. (**e**) Relative viability of MM1R cells treated for 24 h with increasing concentrations of WA, upon BTK silencing (siBTK) in presence or absence of C481S BTK overexpression. Data are plotted as mean ± s.d., *n* = 3 biologically independent replicates (ns = *p* > 0.05, * *p* = 0.0309, *** *p* < 0.0001, ANOVA).

Human protein kinases are composed of two highly conserved domains, namely catalytic and regulatory domains. It has been shown that the catalytic domain can be further classified based on the positions of the gatekeeper amino acids and cysteines hosted in the catalytic pocket [69,70,71,72]. Based on the positions of cysteines present across the human kinome, Leproult et al. reclassified the human kinome into a cysteinome according to the cysteine positions relative to the ATP binding pocket (Table 2) [69,73]. Interestingly, most kinases inhibited by WA treatment, including BTK, BLK and EGFR, were found to be highly enriched in Hinge-6 domain type kinases (Table 2). Multiple sequence alignment of these Hinge-6 domain orthologs revealed the presence of a conserved glycine-cysteine motif, suggesting that WA covalently binds to this site within its TK targets (Figure 5a). Through molecular docking studies, we found that the conserved cysteine site within BTK (Cys481) is indeed accessible and favorable (−4.93 kcal/mol) for WA binding (Supplementary Table S1). The docking results predict that covalent bond formation occurs via the C_4_-OH group of WA and the Cys481 residue of BTK and that this interaction is further stabilized via hydrogen bond formation with surrounding Leu482, Tyr485 and Gly480 amino acids (Figure 5b,c). Given that IBR also covalently targets the Cys481 residue of BTK, these results suggest that WA interacts with BTK in a similar manner as IBR [63]. By using the NanoBRET Target Engagement Intracellular Kinase assay, where the affinity of WA for BTK can be analyzed by competitive displacement of a fluorescent NanoBRET Tracer bound to BTK, we could further confirm that WA can displace the WT BTK protein, but not the mutated C481S BTK protein in HEK-293 cells (Figure 5d). This suggests that the Cys481 residue of BTK is the main binding site of WA. Furthermore, we show that silencing of endogenous WT BTK reduces cell MM1R viability and can be rescued upon overexpression of C481S BTK overexpression. (Figure 5e, Figure S8). Of special note, C481S BTK overexpression cannot completely rescue WA-induced cell death in MM1R, confirming that WA kinase effects on cell viability are not limited to BTK alone and may involve additional hinge 6 domain type kinase targets of the cysteinome classification in MM1R cells. Along the same line, GC-sensitive MM1S cells lacking BTK overexpression are also sensitive to WA treatment through promiscuous covalent cysteine targeting of alternative cell survival tyrosine kinases expressed in MM1S cells (Figure S9a–c).

## 3. Discussion

A common feature of many B-cell malignancies is their ability to develop therapy resistance through increased BCR signaling and BTK activity. Along the same line, increased BCR signaling has been shown to reduce the therapeutic efficacy of GCs in B-cell leukemias [26,27,28,29]. Although MM cells are typically lacking active BCR, BTK overexpression and signaling has been associated with increased therapy resistance in the disease [18,75]. Accordingly, we compared the anticancer effect of the clinically approved BTK kinase inhibitor IBR and the preclinical phytotherapeutic kinase inhibitor WA to kill GC therapy-resistant MM cells via suppression of BTK hyperactivation. Phosphopeptidome based tyrosine kinome profiling confirmed hyperactivation of multiple BCR family kinases besides other receptor TK families (FGFR, PDGFR, DDR) in GC therapy-resistant MM1R cells. Furthermore, hyperactivated BCR kinases could completely be suppressed with both kinase inhibitors, WA and IBR. Nonetheless, kinase tree representations of both kinase inhibitor profiles revealed quantitative and qualitative differences in specificity and potency, which may underly the differences in potency of WA and IBR to kill GC-resistant MM cells. Biotin affinity-purification experiments confirmed a cysteine-dependent covalent interaction between WA and BTK, similar to IBR-BTK Cys481 binding [63]. This is supported by molecular modeling studies, which revealed favorable covalent WA binding to the conserved Cys481 residue in BTK. In addition, NanoBRET Target Engagement Intracellular Kinase assays confirm WA-dependent inhibition of WT BTK kinase activity, which is lost upon C481S mutation. Interestingly, most kinases inhibited by WA treatment, including BTK, BLK and EGFR, belong to the Hinge-6 domain-type kinases, according to the kinase cysteinome classification. The latter suggests broad redundant kinase inhibitory effects of WA through covalent cysteine targeting. Accordingly, BTK silencing experiments could only partially mimic the therapeutic cell death effects of WA in MM1R, whereas overexpression of the C481S kinase mutant could only partially protect against WA. Similarly, cancer therapeutic effects of WA could also be observed in GC-sensitive MM1S cells, which lack BTK overexpression, through covalent targeting of alternative cell survival kinases expressed in MM1S. As such, the therapeutic efficacy of WA against different cancer cell types may strongly depend on its promiscuous nucleophilic cysteine reactivity towards the cellular repertoire of hyperactivated tyrosine cell survival kinases. Additionally, the anticancer effects of WA may also partially rely on its non-covalent interactions with target proteins. H-bond interactions with terminal hydroxyl groups of WA were shown to be sufficient to deform protein complexes through naïve hindrance [65]. Alternatively, the covalent interaction of WA may be weaker than that of the normal covalent bond and be reversible in washout experiments [76]. Taunton and coworkers demonstrated that drug efficacy could be optimized by finetuning warhead residence time and exploiting the intrinsic reversibility of the reaction moiety [66,77]. In this respect, partially reversible covalent binding characteristics of WA may promote more promiscuous tyrosine kinase targeting than IBR and explain the higher efficacy of WA than IBR in overcoming MM drug resistance. Finally, WA-mediated inhibition of BTK kinase activity was also accompanied by a time-dependent decrease in BTK mRNA and protein expression, not observed for IBR, suggesting that WA targets BTK hyperactivation at multiple levels [62]. For example, WA may indirectly decrease BTK activity by reducing kinase protein levels by inhibition of Sp1-dependent transcription [78], by post-transcriptional microRNA silencing mechanisms [53,79], or by decreasing kinase stability via heat shock chaperone proteins [45,47].

Although WA clearly suppresses hyperactivated BTK family kinases signaling, some limitations of our study need to be acknowledged. Most notably, mainly GC therapy-resistant MM cell models were studied here. Further preclinical in vitro and in vivo studies are required to fully appreciate the therapeutic efficacy of WA to overcome multidrug resistance by covalent inhibition of hinge 6 cysteine domain BTK family kinases. In addition, small qualitative and/or quantitative variations in specificity/potency observed for the promiscuous BCR TK inhibitor profiles of WA and IBR may not be sufficient to explain the large difference in the therapeutic efficacy of WA as compared to IBR against GC-resistant MM cell lines. Of special note, whereas nM concentrations of IBR are effective against B-cell lymphoma cancer cells, much higher μM concentrations seem to be required to kill BTK overexpressing MM cells [17,80,81,82,83], presumably because of the presence of highly redundant BCR/NFKB survival pathways (PI3K/Akt/mTOR/Syk) in MM, which compensates for pharmacological BTK inhibition [37]. Yet, despite the presence of these compensatory pathways, complete silencing of BTK expression is cytotoxic to MM1R cells, emphasizing BTK’s involvement in myeloma cell survival. This is in line with previous observations made in BTK KO mouse models, where complete loss of BTK expression resulted in a more significant decrease in splenic B-cell numbers compared to mice harboring BTK mutations [84]. Because BTK also possesses crucial shuttling and scaffold activities, complete silencing of BTK is likely more detrimental to B-cells due to additional loss of non-kinase functions [85].

Furthermore, covalent cysteine binding of WA has also been reported to Ser/Thr kinases [86] and phosphatases [87], which were not included in our phosphopeptidome screening approach. Indeed, in addition to BTK, BCR signaling is fine-tuned by Ser/Thr kinases (for example, IKK2, PKCβ) [15,88] and phosphatases (i.e., SHIP1, PTEN) [89,90]. Finally, chemoproteomic strategies have identified additional WA non-kinase target proteins, which may further strengthen the potential cancer therapeutic efficacy of WA [45,91]. Therefore, functional silencing approaches with shRNA libraries will be informative to further distinguish druggable key cancer targets from adverse off-targets to defeat therapy resistance in MM by WA [87,92]. Further research determining the safety and pharmacokinetic profile of WA is needed as well. Preliminary (pre)clinical toxicology studies of WA in different cancer models revealed that WA administration is generally well tolerated with limited to no adverse toxicity reported [93,94,95]. Yet, no toxicology data of WA have been collected in B-cell malignancies, such as MM.

## 4. Materials and Methods

### 4.1. Cell Culture and Cell Viability Assays

GC-sensitive MM1S (CRL-2974) and GC-resistant MM1R MM cell lines (CRL-2975) have been described previously and were purchased from ATCC [53]. GC-resistant U266 cells were kindly provided by Dr. Eva Lion, Head of Tumor Immunology Group of the Laboratory of Experimental Hematology (University of Antwerp). Cells were cultivated in RPMI-1640 supplemented with 10% fetal bovine serum (E.U approved; South American origin) and 1% penicillin/streptomycin solution (Invitrogen, CA, USA). The cell lines were additionally supplemented with 1% MEM Non-Essential amino acids and 1% sodium pyruvate (Invitrogen, CA, USA). Each cell line was maintained at 37 °C in 5% CO_2_ and 95% air atmosphere and 95–98% humidity. Cell viability was assessed by colorimetric assay with 3-(4, 5-dimethylthiozol-2-yl)-2, 5-diphenyltetrazolium bromide (MTT) (Sigma-Aldrich, St. Louis, MO, US) as previously described [53].

### 4.2. Cell Lysis and Peptide Array-Based TK Activity Profiling

WA- or BTK-treated and untreated MM1S and MM1R cells (*n* = 3 biologically independent samples per cell line per treatment) were lysed in M-PER lysis buffer containing 1:100 Halt’s protease and Pierce™ phosphatase inhibitors (Thermo Scientific, Waltham, MA, USA). The lysates were collected and centrifuged at >13,000× rpm for 15 min at 4 °C. The supernatants were transferred to pre-chilled Eppendorf tubes and flash-frozen immediately on dry ice. Protein concentrations were determined by the Pierce™ BCA method (Thermo Scientific, Waltham, MA, USA) [96]. Cellular kinase activities were measured by TK-specific phosphopeptide arrays (PamChip, PamGene International B.V. the Netherlands) according to the manufacturer’s protocol as previously described [56,97]. The differences in peptide phosphorylation signal intensity between different experimental setups were analyzed in a linear mixed model by using BioNavigator 6.3 software integrated with R (Bioconductor) for statistical analysis (paired two-sided t-test and unpaired *t*-tests). FDR were calculated using Benjamini–Hochberg method (adjusted *p*-values < 0.05) [55]. The upstream kinases were correlated with the identified PTK-specific phosphopeptide fingerprints according to the human (phospho)protein reference database [98,99,100,101]. Peptide lists showing differential phosphorylation intensities were further cross-compared with phosphorylation specific datasets of the Kinexus kinase predictor [102]. The kinases’ ranking was based upon scoring penalties of sensitivity and specificity of observed peptide phosphorylation changes in the tested experimental conditions. The significance of the upstream kinase scoring functions was calculated by Fisher’s exact test. Pathway enrichment of differentially phosphorylated protein IDs was analyzed via MetaCore (Clarivate Analytics, Philadelphia, PA, USA) and Ingenuity pathway analysis (IPA) software (Qiagen, Germany) [103].

### 4.3. cDNA Conversion Quantitative Real-Time PCR

Total RNA (1 µg) extracted from each sample was converted into cDNA with the Go Script reverse transcription system (Promega, Madison, WI, USA) following the manufacturer’s protocol. Next, qPCR analysis was carried out using the GoTaq qPCR master mix (Promega, Madison, WI, USA) according to the manufacturer’s instructions. In brief, a 25 µL reaction volume mix per sample was prepared, containing 12.5 µL GoTaq qPCR master mix, 0.4 µM forward and reverse primer and nuclease-free water. The following PCR program was applied on the Rotor-Gene Q qPCR machine of Qiagen: 95 °C for 2 min, 40 cyclic denaturations (95 °C, 15 s) and annealing/extension (60 °C, 30 s), and dissociation (60–95 °C). Each sample was run in triplicate. The median value of the triplicates was taken to calculate the ΔΔCt-values using GAPDH as the normalization gene. Primer sequences are listed in Supplementary Table S2.

### 4.4. RNA Extraction and RNA Sequencing

The RNeasy mini kit (Qiagen, Germany) was used to extract total RNA from untreated, or WA exposed MM1S and MM1R cells (*n* = 3 biologically independent samples per cell line per treatment) according to the manufacturer’s protocol. Isolated, pure total RNA was then quantified and qualified using an Epoch^TM^ microplate spectrophotometer (BioTek, Winusky, VT, USA). RNA samples were stored at −80 °C and subsequently shipped to BGI (BGI Group, Beijing, China), where RNA integrity was determined using the 2100 Bioanalyzer system (Agilent Technologies, CA, USA). All 12 samples with acceptable quality levels (RNA content >80 ng/µL, 28 s/18 s ≥ 1.0 and RIN ≥ 7.0) were included for sequencing library preparation. Prepared libraries were 2 × 50 bp paired-end sequenced using the BGISEQ-500 platform (BGI Group, China). RNAseq data were deposited in the NCBI GEO database (GSE162475).

The quality of the RNA sequencing reads was assessed using FastQC (v0.11.5, Babraham Institute, Cambridge, UK) [104]. STAR (v2.7.3a, Cold Spring Harbor Laboratory, CA, USA) [105] was subsequently used to map reads to the human reference genome build 37 (hg19) and to generate a read count table summarizing the counts per gene. Finally, differential gene expression analysis was performed using the DESeq2 package [57], and pathway analysis was performed with Panther (Protein Informatics, Celera Genomics, CA, USA) [106].

### 4.5. Antibodies and Reagents

WA was purchased from Alta Vista Phytochemicals (Hyderabad, India), and biotinylation of WA was performed by Dr. P. Van der Veken (WA-BT; Universiteit Antwerpen, Belgium). Both formulations were stored as 20 mM stocks in DMSO at −20 °C as previously described [48,107]. IBR (IMBRUVICA^®^) stock solutions were obtained from Pharmacyclics (Sunnyvale, CA, USA). Antibodies BTK (3533) and GAPDH (2118S) were obtained from Cell Signaling Technology (Danvers, MA, USA).

### 4.6. Cell Viability after WA Washout

After 15 min treatment of MM1R cells with increasing concentrations of WA (with or without DTT), cells were washed extensively with PBS (3 × 5 min) and left to grow for an additional 24 h in WA-free supplemented RPMI-1640 medium. Once incubation was complete, cell viability was determined by colorimetric MTT assay as described above. MM1R cells continuously treated with WA for 24 h were included to allow for a direct comparison of covalent and non-covalent effects on cell viability.

### 4.7. Protein Extraction and Western Immunoblot Analysis

For western immunoblot analyses, cell pellets were lysed in 0.5 mL RIPA buffer (150 mM NaCl, 0.1% Triton X-100, 0.1% SDS, 50 mM Tris-HCl pH 8) supplemented with protease inhibitors (Complete Mini^®^, Roche, CA, USA). Soluble protein extracts were obtained after 15 min incubation on ice, followed by brief sonication and centrifugation at 16 g for 20 min at 4 °C. Samples were separated by sodium dodecyl sulfate-polyacrylamide gel electrophoresis (SDS–PAGE) and transferred onto nitrocellulose membranes (Hybond C, Amersham) following standard protocols. After blocking, membranes were incubated overnight at 4 °C with the primary antibodies, followed by dye-conjugated secondary antibodies (polyclonal goat anti-rabbit HRP, #P0448, Dako). Bound complexes were detected with the Amersham Imager 680 (Cytiva, Austria) and quantified by Image J software (Max Planck Institute, Dresden, Germany) [108]. Original immunoblot images are provided in Supplementary Table S4.

### 4.8. WA-Biotin-Based Affinity Purification

WA-BT affinity purification and co-precipitation were performed as previously described [48]. In short, MM1R and U266 cells were seeded in 10 cm culture dishes and incubated for 24 h at 37 °C. Cells were treated with biotinylated WA (1 µM, 2.5 µM, 5 µM or 7.5 μM as indicated) or left untreated for 2 h. Cells were then lysed in 1 mL lysis buffer (5 mM Tris pH 7.6, 1% Triton-X100 and 5 mM EDTA, supplemented with Complete™ protease inhibitor cocktail) and incubated with Neutravidin beads overnight. Beads were centrifuged for 5 min at 500 g after which the supernatant was removed. Beads were subsequently washed 5 times with lysis buffer. Total protein lysates, or coprecipitates, were separated by SDS–PAGE and electrotransferred onto a nitrocellulose membrane. Blots were probed using the appropriate antibodies, and the immunoreactive proteins were detected using the Amersham Imager 680 (Cytiva, Austria).

### 4.9. Immunofluorescence Confocal Microscopy

After treatment with WABI, MM1R cells were fixed in 4% (*v*/*v*) formaldehyde. Cells were stained with rhodamine–phalloidin (Molecular Probes, Thermo Fisher Scientific Inc., Waltham, MA, USA) to visualize the cellular morphology, Hoechst 33,258 (Sigma-Aldrich, St. Louis, MO, USA) to visualize the nucleus and streptavidin-Alexa Fluor555 (Molecular Probes, Thermo Scientific, MA, USA) to visualize biotinylated WA. To determine WABI colocalization with specific proteins (BTK), WABI-treated MM1R cells were immunostained with primary (anti-rabbit BTK, Cell Signaling Technology, mAb#8547) and corresponding labeled secondary antibodies (anti-rabbit Alexa Fluor-488, Molecular Probes, Thermo Scientific, MA, USA). Cells were then placed on glass slides and mounted with coverslips using Fluoromount^TM^ (Sigma-Aldrich, St. Louis, MO, US)Confocal imaging was carried out by a laser-scanning microscope equipped with a Plan-Apochromat 63X/1.40 Oil DIC objective lens and excitation wavelengths 405, 488, 561 and 640 nm (Zeiss LSM 800, Carl Zeiss, Germany). At least 20 different microscopic fields were analyzed for each sample using ZEN imaging software (Carl Zeiss). ZEN lite^TM^ (Carl Zeiss, Germany) was used to perform image reconstruction and presentation.

### 4.10. Covalent Docking of WA with BTK

In silico molecular docking studies of the BTK protein (PDB id: 6TFP) with WA (PubChem CID: 26537) were performed as previously described [109]. Briefly, BTK and WA structures were energy minimized with Swiss-PdbViewer (v4.1) [110] and UCSF Chimera (Geneva Biomedical Institute, Geneva, Switzerland) [111], respectively. Molecular docking and calculation of the docking scores were performed with Autodock4 (v4.2.6, University of California, Berkeley, CA, USA). The obtained docking solutions were evaluated based on their scoring, and the generated poses were clustered based on the ligand’s cluster ranks. Finally, the clusters were differentiated based on the covalent bond lengths and prime energies. Final docking results were visualized with LIGPLOT (v.4.5.3, University College London, UK) [74] and PyMOL (v.2.4, Schrödinger, NY, USA) [112] software.

### 4.11. Cell Transfections and BRET Measurements

BTK- and BTK(C481S)-NanoLuc^®^ fusion vectors were purchased from Promega and used in BRET target engagement experiments. HEK-293 cells were transfected with the NanoLuc^®^ fusion vectors using FuGENE HD (Promega, Madison, WI, USA) according to the manufacturer’s protocol. Briefly, fusion vectors were diluted into transfection carrier DNA (Promega, Madison, Wisconsin, USA) at a mass ratio of 1:10, after which FuGENE HD was added at a ratio of 1:3. The FuGENE HD complexes were then added to HEK-293 cells (1:20 ratio) at a density of 2 × 10^5^ per mL. Subsequently, cells were plated onto white, 96-well plates (Corning, NY, USA) at a density of 2 × 10^4^ cells/well and left to incubate for 24 h at 37 °C and 5% CO_2_. After incubation, cells were equilibrated for 2 h with the NanoBRET tracer reagent (K11, Promega, Madison, WI, USA) and increasing WA concentrations (0.5–10 µM). To measure BRET, NanoBRET NanoGlo substrate and extracellular NanoLuc inhibitor (Promega, Madison, WI, USA) was added according to the manufacturer’s protocol, and filtered luminescence was measured on a GloMax Discover luminometer equipped with 450 nm BP filter and 600 nm LP filter. MilliBRET units (mBU) were calculated by multiplying raw BRET values by 1000 and plotted with GraphPad Prism.

### 4.12. Nucleofection of siBTK and C481S BTK

The BTK(C481S)-NanoLuc^®^ Fusion Vector was purchased from Promega, and siBTK was purchased from GE-Healthcare Bio-sciences (Accell Human BTK siRNA, EQ-003107–00–0005, 29121299, sequence supplied in Table S3). MM1R cells were transfected with siBTK alone or siBTK combined with BTK(C481S) using the Nucleofector IIb device (Lonza, Switzerland) according to the manufacturer’s instructions. In short, 2.10^6^ MM1R cells were resuspended in supplemented nucleofector solution, after which 300 nM siBTK was added with or without 2 µg BTK(C481S). To each nucleofection reaction, an additional 2 µg of pmaxGFP™ Vector was added as an internal positive control (transfection efficiency = 51.3 ± 2.4%). Resuspended cells were subsequently transferred to a cuvette and transfected using the O-020 nucleofector program. After nucleofection, a pre-equilibrated medium was added, and cells were transferred to a 96-well plate at a density of 80.000 cells/well. Eight hours post-transfection, transfection efficiency and GFP expression were assessed with fluorescence microscopy. If a GFP signal was present, cells were treated with increasing concentrations of WA and cell viability was measured after 24 h using the MTT colorimetric method. BTK mRNA expression of nucleofected cells was further confirmed by quantitative real-time PCR.

## 5. Conclusions

Although cysteine code classification has been developed to explain the selectivity of different electrophiles classes, many cysteine-reactive natural products have shown promiscuous therapeutic anticancer activities with poorly characterized targets [87,113,114]. In a high-throughput compound library screening, the cysteine reactive phytochemical WA was identified as a top prioritized investigational compound to suppress hyperactivated BCR cancer signaling, together with IBR [63]. Given that WA possesses broad-spectrum therapeutic activities in several (drug-resistant) cancer cell types, including B-cell lymphoma and MM [46,51], we further characterized its molecular mechanisms of action in GC-resistant MM cells by combining innovative transcriptomic and phosphor-peptidomic kinase (activity) profiling approaches. Our findings demonstrate that the TK inhibitory profile of WA in GC-resistant MM cells is similar to that of IBR and that hyperphosphorylation of many BCR-related kinases, including BTK, is inhibited. Because WA also covalently targets BTK in a cysteine-dependent manner, we hypothesize that WA inhibits BTK autophosphorylation and activation in an IBR-like fashion. We additionally found that WA silences BTK expression in GC-resistant MM cells at the mRNA and protein level, suggesting that WA suppresses BTK cancer signaling via a dual mechanism. Since WA displays high a similarity with IBR mode of action, but with a more potent effect on cell death of GC-resistant MM cells, further structure-function analysis of WA-analogs may allow (pre)clinical optimization of highly effective withanolide BCR inhibitors to expand the arsenal of cancer drugs against therapy-resistant B-cell malignancies.

## Figures and Tables

**Table 1:** Overview of top 20 most significantly differentially expressed genes (logFC > 1 or logFC < −1) between glucocorticoid (GC)-resistant MM1R and GC-sensitive MM1S cells.

Gene Ontology Term	Symbol	Gene ID	Name	Log2FC	*p*-Adj.
ECM and cell–cell adhesion	RELN *	5649	Reelin	2.9	7.4 × 10^−160^
	PLXNB2	23654	Plexin B2	1.8	7.1 × 10^−96^
	PODXL2 *	50512	Podocalyxin like 2	2.6	6.8 × 10^−83^
	ESAM	90952	Endothelial cell adhesion molecule	2.0	1.7 × 10^−80^
	PRKX *	5613	Protein kinase X-linked	1.1	8.1 × 10^−79^
	ACP5	54	Acid phosphatase 5, tartrate-resistant	4.8	2.0 × 10^−77^
GPCR signaling	GNG7	2788	G protein subunit gamma 7	1.5	5.7 × 10^−99^
	UTS2R	2837	Urotensin 2 receptor	1.9	5.4 × 10^−77^
BCR signaling	BTK *	695	Bruton’s tyrosine kinase	2.8	1.5 × 10^−216^
	TNFRSF8	943	TNF receptor superfamily member 8	3.7	6.9 × 10^−93^
	CD52	1043	CD52 molecule	3.5	5.6 × 10^−83^
mRNA/protein stability	CTAG2	30848	Cancer/testis antigen 2	8.5	4.0 × 10^−168^
	LINC01518	101929397	Long intergenic non-protein Coding RNA 1518	7.9	1.4 × 10^−147^
	CMTR1	23070	Cap methyltransferase 1	−1.1	9.6 × 10^−115^
	TMEM25	84866	Transmembrane protein 25	4.8	6.0 × 10^−79^
Cell cycle regulation	CDKN2A	1029	Cyclin-dependent kinase inhibitor 2A	9.4	1.7 × 10^−210^
Cytoskeleton	TUBB4A	10382	Tubulin beta 4A class IVa	3.6	6.1 × 10^−165^
Inflammation	NLRP11	204801	NLR family pyrin domain containing 11	4.9	1.2 × 10^−220^
Transmembrane transport	SLC38A5	92745	Solute carrier family 38 member 5	1.8	1.7 × 10^−92^
	ABCG2*	9429	ATP-binding cassette subfamily G member 2	5.6	2.0 × 10^−84^

* Genes associated with therapy resistance. Abbreviations: ECM, extracellular matrix; GPCR, G-protein coupled receptor; BCR, B-cell receptor.

**Table 2:** Kinase cysteinome classification. Summarized from [73]. Bold highlights kinases representing the main tyrosine kinase targets of withaferin A.

Site	Subsite	Representative Kinases
Gatekeeper region	GK	MOK
	GK + 1	SgK494
	GK − 1	MAP2K4, MKK3, MAP2K6, KHS1, KHS2, GCK
DFG region	DFG + 1	MAP3K8, MOS, MAP3K4, PINK1
	DFG + 2	PKCz, PKCi, AKT1, AKT2, AKT3, PKCg, SGK1F, SGK2
	DFG − 1	PBK, TGFbR2, CDKL3, CDKL2, PRP4, MNK2, MNK1
Glycine rich loop region	Glycineloop	WNK4, WNK1, WNK2, WNK3, HER3
	Glycineloop 1	ZAK
	Glycineloop 2	SgK496, MEKK1, PLK2, PLK3, PLK1, RSK1
	Glycineloop 3	SgK493
	Glycineloop5	FGFR1, FGFR2, FGFR3, FGFR4
Hinge binding region	Hinge 1	FGFR4, TTK, MAPKAPK2, MAPKAPK3
	Hinge 2	IKKa, IKKb, LKB1, NEK4, Wee1, SLK, FLT4, KDR
	Hinge 3	Ron, FGR, SgK494, Kit, CSFR, FLT3
	Hinge 4	SgK110, BubR1, LKB1, TBK1
	Hinge 5	PINK1, EphB3
	Hinge 6	MAP2K7, TEC, TXK, ITK, BTK, BMX, BLK, HER2, EGFR, HER4, JAK3
	Hinge 7	JNK1, JNK2, JNK3
Roof region	Roof sheet	HER3

## Data Availability

The data presented in this study are openly available in the NCBI GEO database, GEO accession number GSE162475.

## Supplementary Materials

The following are available online at https://www.mdpi.com/article/10.3390/cancers13071618/s1, Figure S1: Heatmap showing phosphorylation intensities of peptides serving as substrates for tyrosine kinases. Figure S2: MetaCore pathway analysis of differentially phosphorylated protein peptides (*p* < 0.05) in MM1R versus MM1S cells. Figure S3: Row-annotated heatmap representation of hyperactivated or inhibited kinases in MM1R versus MM1S cells. Figure S4: Target-binding affinities of Ibrutinib. Figure S5: Relative viability of U266 cells after 24 h treatment with WA or DEX. Figure S6: Relative BTK mRNA and protein levels of MM1R cells treated with IBR. Figure S7: Relative viability of MM1R cells treated with increasing concentrations of WA for 15 min. Figure S8: Relative BTK mRNA and protein expression of siBTK-transfected MM1R cells with or without the C481S BTK mutant. Figure S9: The biological targets of WA treatment strongly depends on cellular context. Table S1: Summary of the binding energy of the covalent bond of Cys481 with WA as predicted by covalent docking. Table S2: Overview of qPCR primers used in this study. Table S3: Overview of siRNA sequences used in this study. Table S3: Overview of siRNA sequences used in this study. Table S4: Original images western immunoblots.
