# Supplementary material for: Covalent Cysteine Targeting of Bruton’s Tyrosine Kinase (BTK) Family by Withaferin-A Reduces Survival of Glucocorticoid-Resistant Multiple Myeloma MM1 Cells
Source: Cancers (Basel). 2021 Mar 31;13(7):1618. doi: 10.3390/cancers13071618 (PMC8037275; doi:10.3390/cancers13071618)
Supplement: Supplementary file 1 [file cancers-13-01618-s001.pdf]

Supplementary Materials

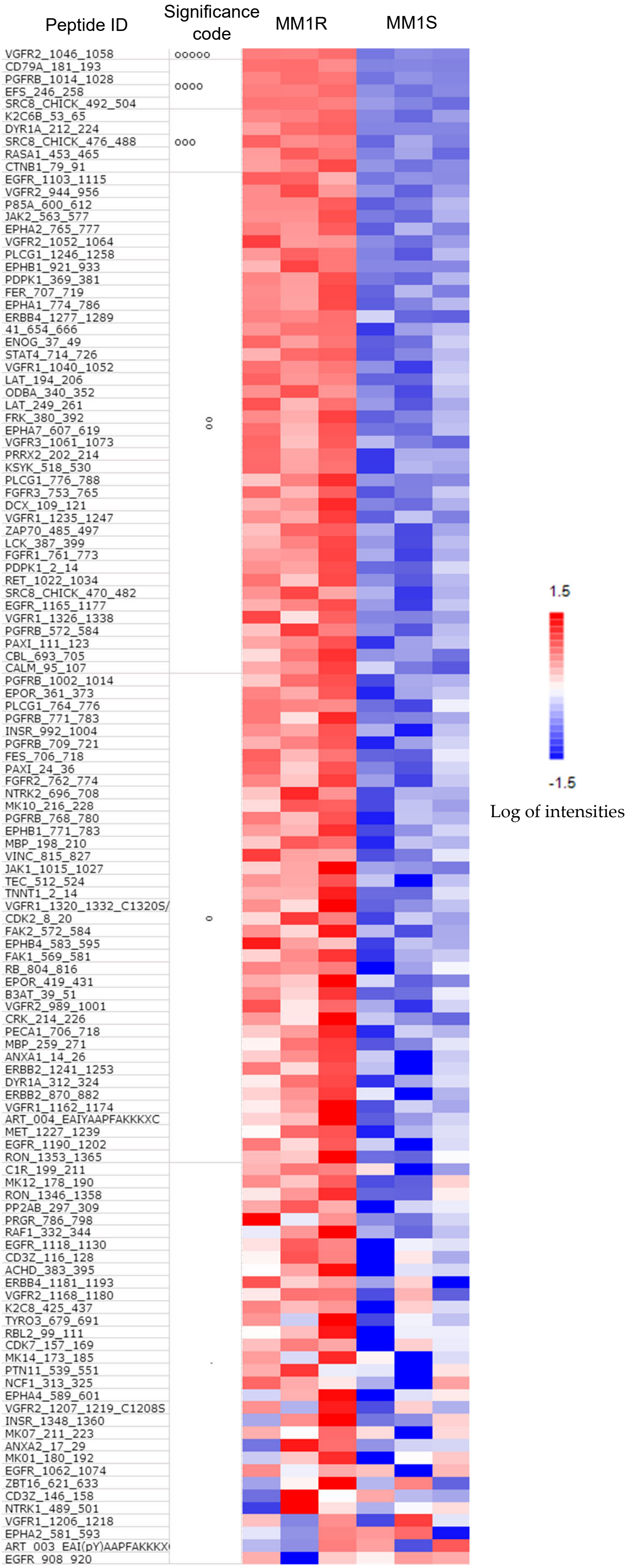

Figure S1 (previous page). Heatmap showing phosphorylation intensities of peptides serving as substrates for tyrosine kinases. Figure shows hyperphosphorylated (red) or hypo phosphorylated (blue) peptides in MM1R (n=3) and MM1S (n=3) samples ranked by the p-value resulting from a t-test. The significance is indicated using a significance code: ooooo: p< 0.00001, oooo: p< 0.0001, ooo: p< 0.001, oo: p< 0.001, o: p< 0.05.

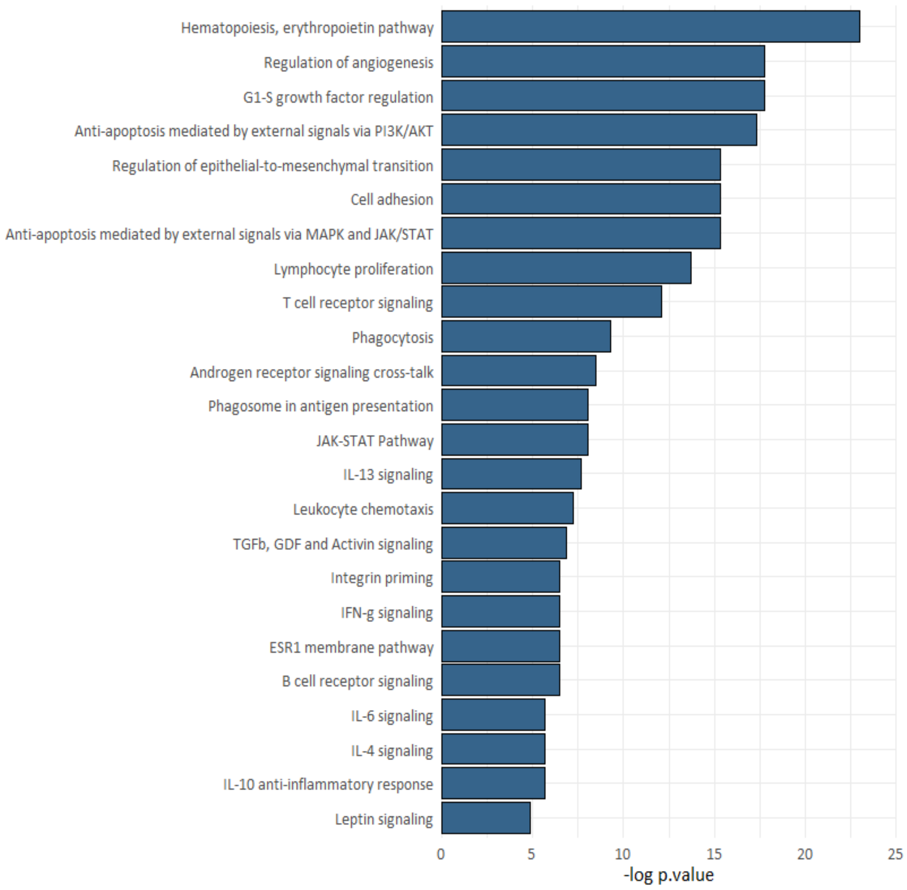

Figure S2. MetaCore pathway analysis of differentially phosphorylated protein peptides (p<0.05) in MM1R versus MM1S cells.

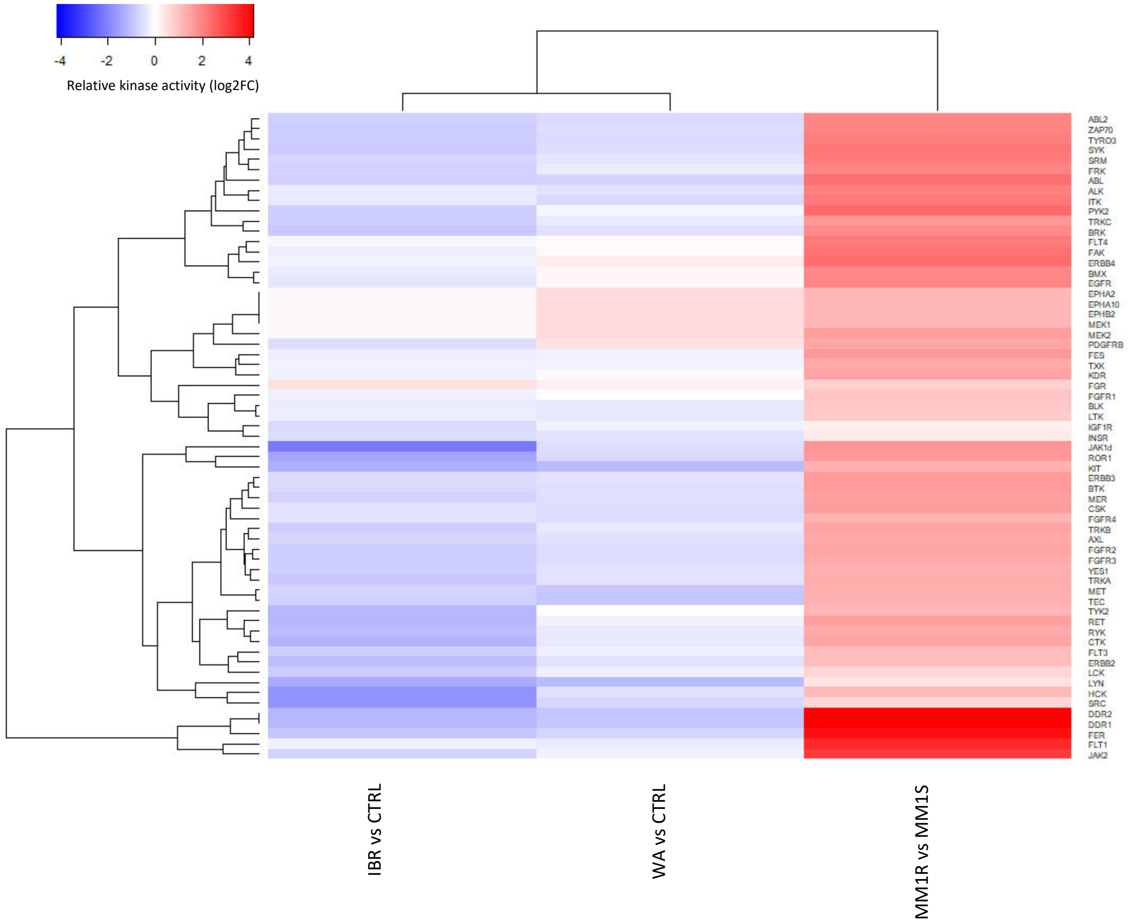

Figure S3. Row-annotated heatmap representation of hyperactivated or inhibited kinases in MM1R versus MM1S cells, or following 15 min IBR or WA treatment of MM1R cells, *n*=3 biologically independent samples per treatment group.

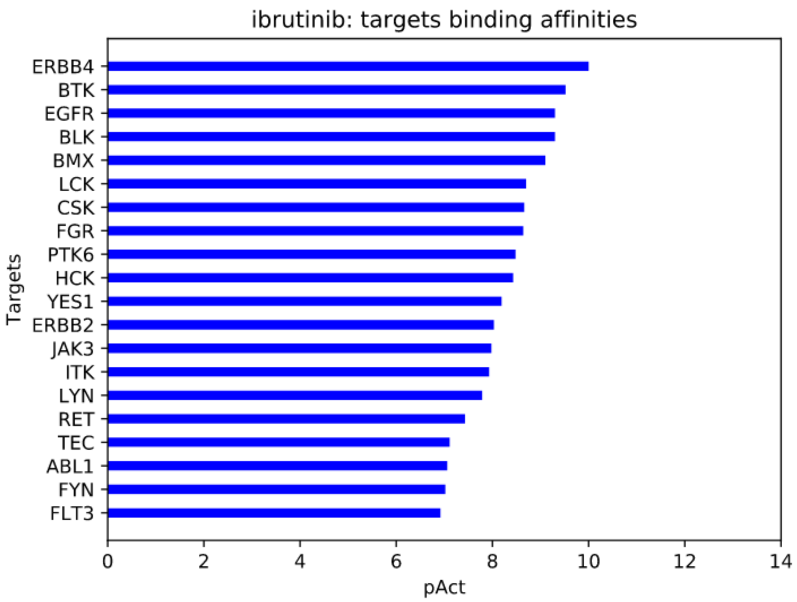

Figure S4. Target binding affinities of Ibrutinib. Binding affinities of Ibrutinib to its targets are shown as pAct (-Log(IC50/Ki/EC50,…)). Bars show the target interactions confirmed by ChEMBL/PubChem/papers. Graph was generated using the drug network map tool of the UC San Diego Abagyan Lab (http://ruben.ucsd.edu/dnet/).

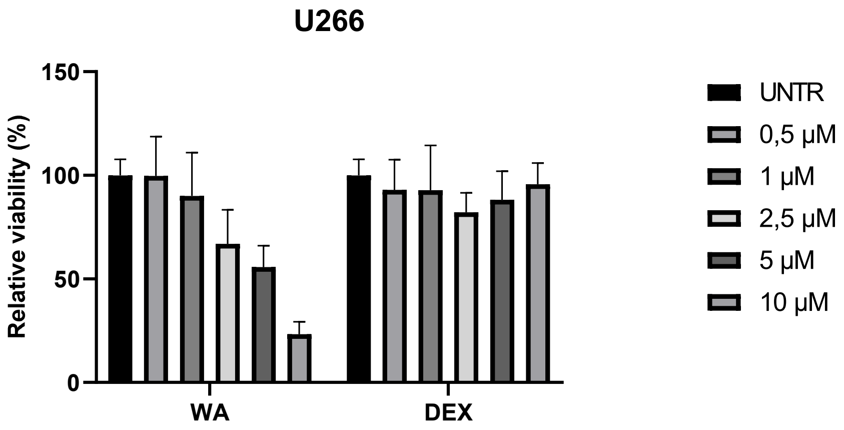

Figure S5. Relative viability (%) of U266 cells after 24 h treatment with WA or DEX. Data are plotted as the mean ± s.d., *n*=3 biologically independent samples.

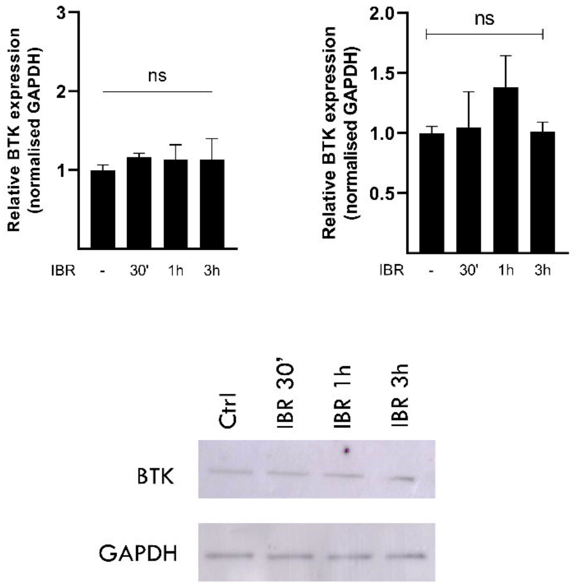

Figure S6. Relative BTK mRNA (upper left) and protein levels (upper right and lower panel) of MM1R cells treated with 1 μM IBR for the indicated timepoints. Data are plotted as mean ± s.d., n=3 biologically independent replicates (ns = p > 0.05, ANOVA).

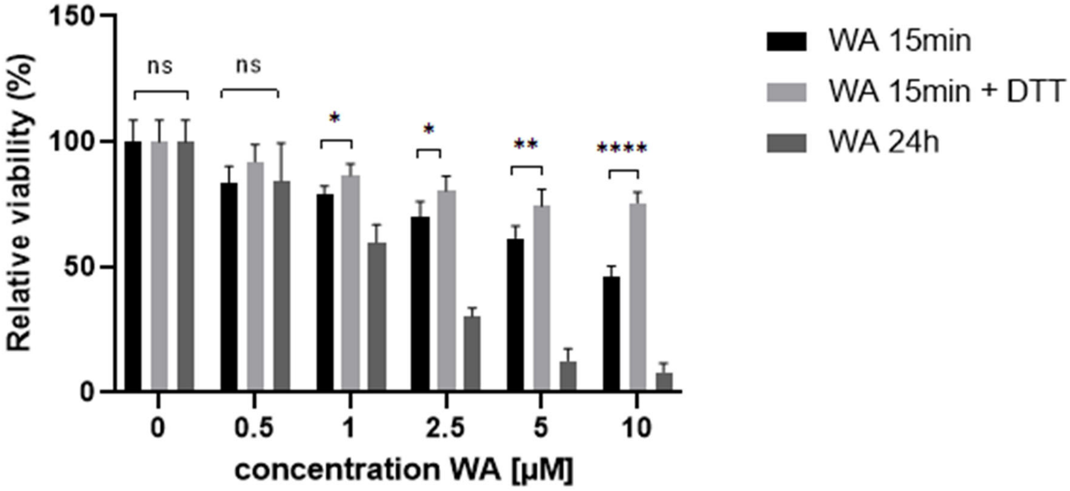

Figure S7. Relative viability of MM1R cells treated with increasing concentrations of WA 24 hrs post treatment. Cells were incubated with WA for 15 min (with or without DTT) after which WA was washed away or continuously for 24 hrs. Data are plotted as mean ± s.d., *n*=3 biologically independent replicates (ns = p > 0.05, *p < 0.05, **p < 0.01, ****p < 0.0001, ANOVA).

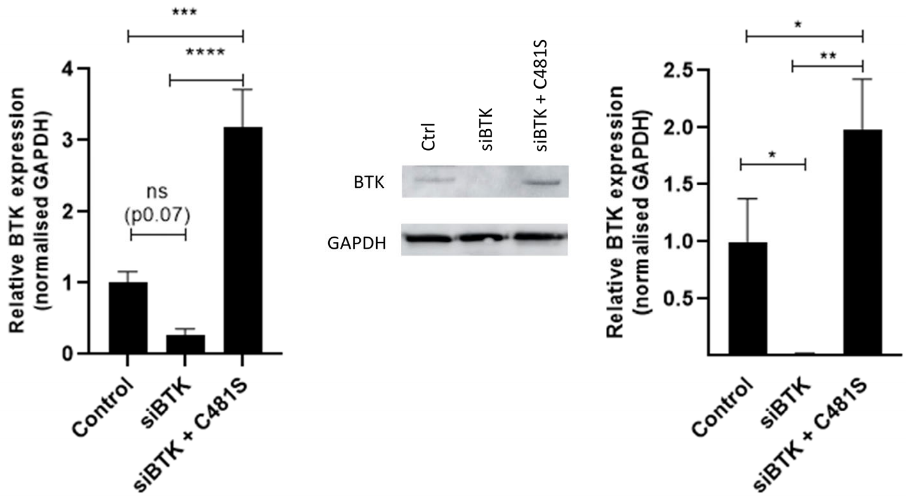

Figure S8. Relative BTK mRNA (left) and protein (right) expression of siBTK-transfected MM1R cells with or without the C481S BTK mutant. Data are plotted as mean ± s.d., n=3 biologically independent replicates (ns = p > 0.05, *p < 0.05, **p < 0.01, ****p < 0.0001, ANOVA).

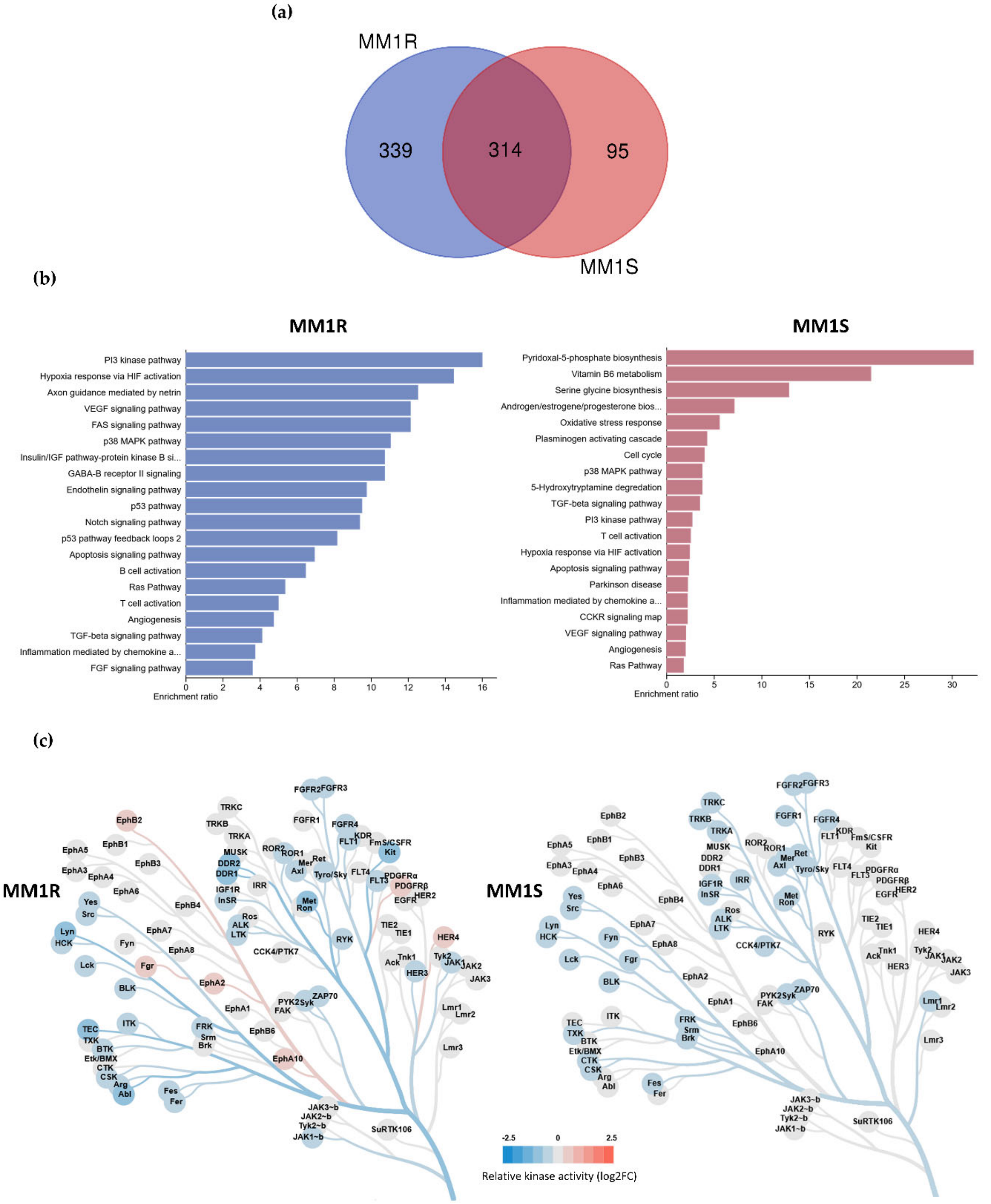

Figure S9. The biological targets of WA treatment strongly depends on cellular context. (**a**) Venn diagram showing the number of significantly differentially expressed genes ($p < 0.05$, logFC < |1|) in MM1R and MM1S cells after WA treatment. (**b**) Panther pathway analysis of significantly differentially expressed genes in MM1R only (left) and MM1S only (right). (**c**) Kinase trees displaying relative changes in kinase activities upon WA treatment in MM1R cells (left) and MM1S cell (right). Kinase trees were generated with the CORAL web tool (http://phanstiel-lab.med.unc.edu/CORAL/)

Table S1. Summary of binding energy and distance of the covalent bond of Cys481 with WA as predicted by covalent docking.

BTK interaction site	PDB ID	Binding energy	Bond length	No. of hydrogen bonds	Amino acid interaction
Cys481	6TFP	-4.93 kcal/mol	1.4 Å	3	Tyr485, Leu482, Gly480

Table S2. Overview of qPCR primers used in this study

Target		Primer Sequence	Gene accession number
BTK	Forward	GTC CCA CCT TCC AAG TCC TG	ENSG00000010671
	Reverse	GCC TCT TCT CCC ACG TTC AA	
GAPDH	Forward	GCT CTC TGC TCC TCC TGT TC	ENSG00000111640
	Reverse	ACG ACC AAA TCC GTT GAC TC	

Table S3. Overview of siRNA sequences used in this study

Target	Target Sequence	siRNA sequence
BTK	UUU UGA UGU GGG AAA UUU A	TAA ATT TCC CAC ATC AAA A

Table S4. Original images immunoblot analysis.

Figure	Blot(s) used in figure	Blot(s) with molecular marker
**1f**: western immunoblot detection and quantification of basal BTK and GAPDH protein levels in MM1R and MM1S	α-BTK* (±77 kDa) and α-GAPDH+ (±37 kDa) 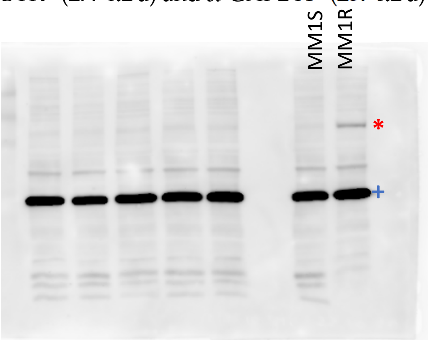	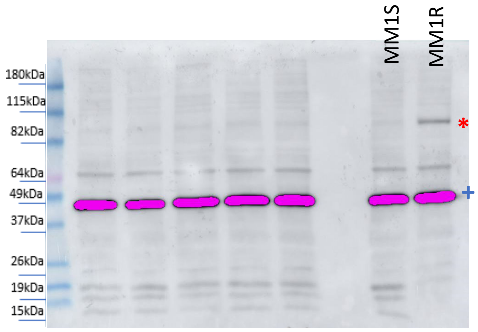
**3c (left):** western immunoblot detection and quantification of BTK and GAPDH expression levels after WA treatment in MM1R cells	α-BTK (±77 kDa) 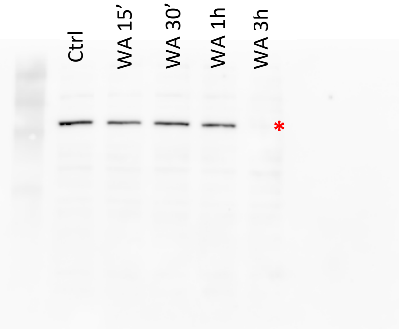 α-GAPDH (±37 kDa) 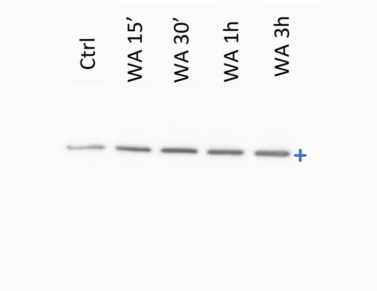	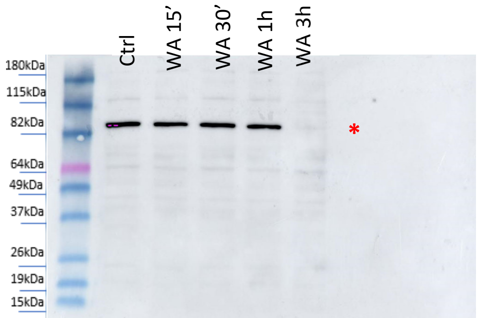 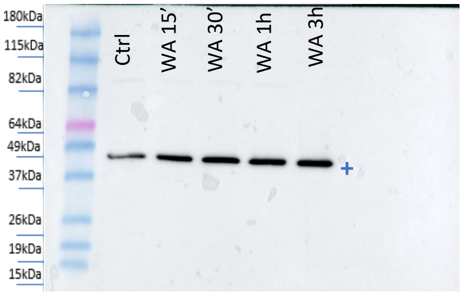
**3c (right):** western immunoblot detection and quantification of BTK and GAPDH expression levels after WA treatment in U266 cells	α-BTK (±77 kDa) 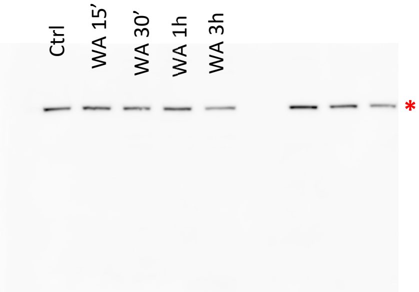	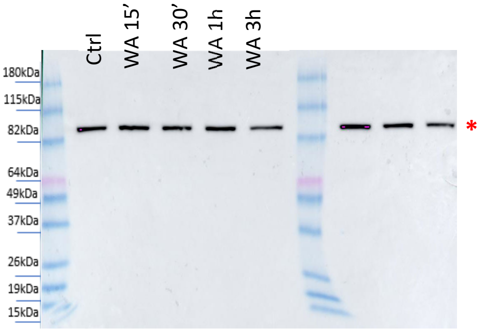

<table>
<tr>
<td></td>
<td>α-GAPDH (±37 kDa)
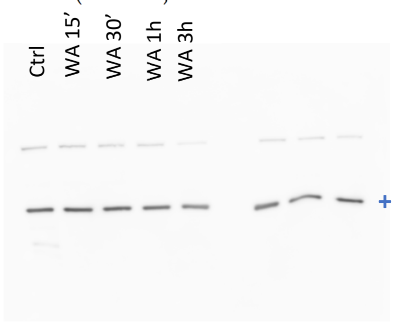</td>
<td>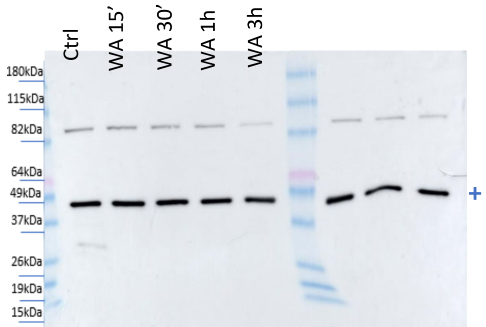</td>
</tr>
<tr>
<td>4a: western immunoblot detection of BTK levels before and after pulldown with biotinylated WA, following 2 h WABI treatment in MM1R cells in the presence or absence of excess thiol donor DTT (1mM)</td>
<td>α-BTK (±77 kDa) – pulldown
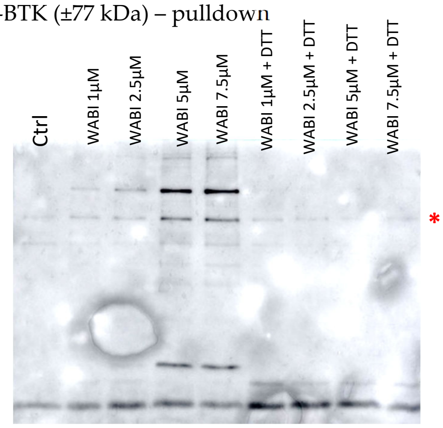
α-BTK (±77 kDa) - input
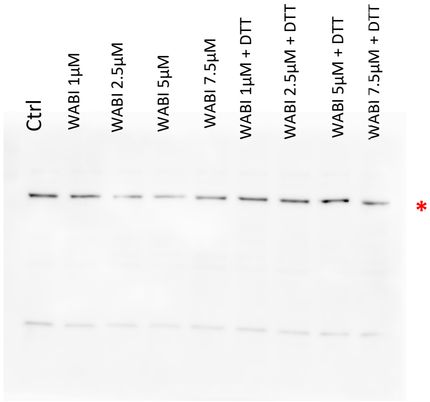</td>
<td>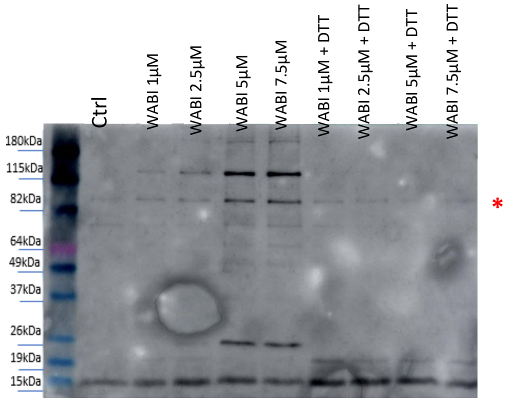
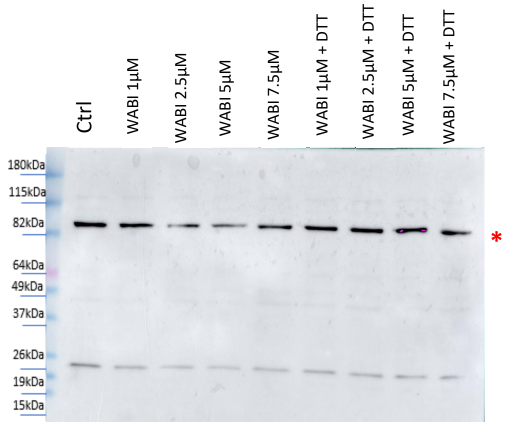</td>
</tr>
<tr>
<td>4b: western immunoblot detection of BTK levels before and after pulldown with biotinylated WA, following 2 h WABI treatment in MM1R cells in</td>
<td>α-BTK (±77 kDa) - pulldown
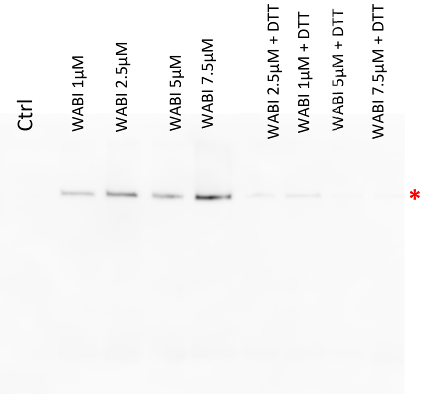</td>
<td>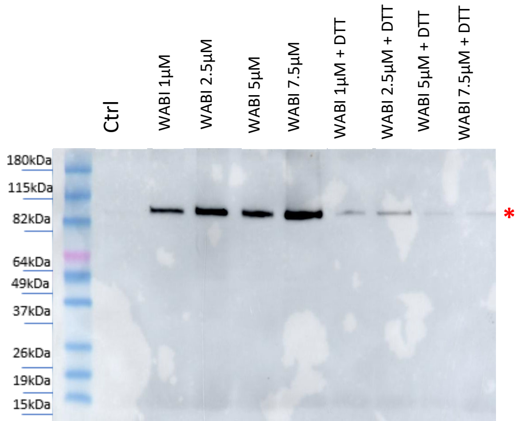</td>
</tr>
</table>

the presence or absence of excess thiol donor DTT (1mM)	α-BTK (±77 kDa) - input 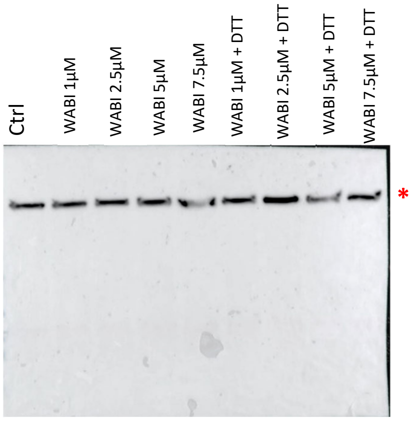	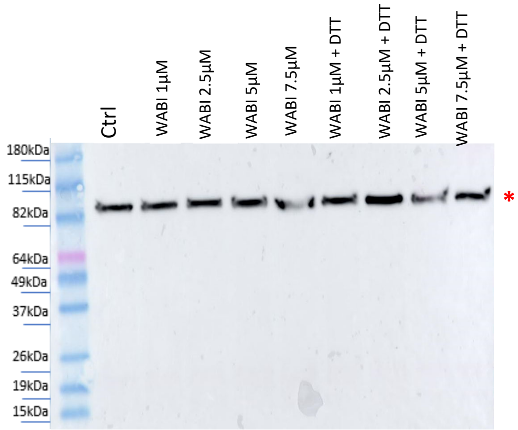
**S6:** western immunoblot detection and quantification of BTK and GAPDH expression levels after IBR treatment in MM1R cells	α-BTK (±77 kDa) 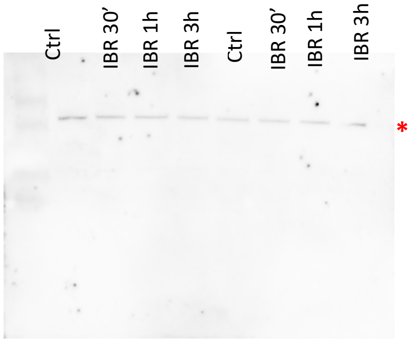 α-GAPDH (±37 kDa) 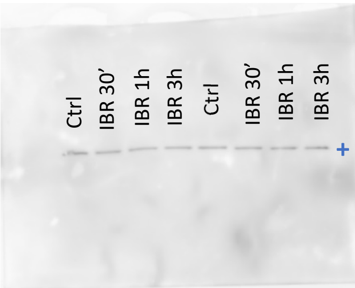	α-BTK (±77 kDa) 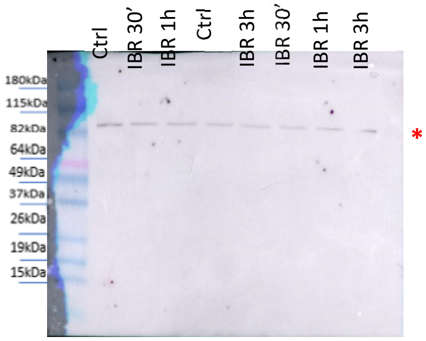 α-GAPDH (±37 kDa) 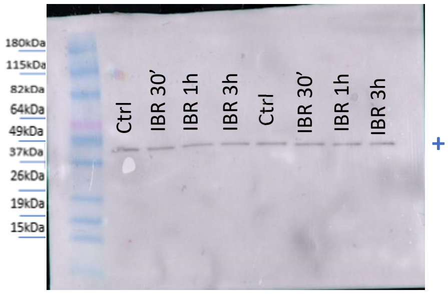
**S8:** western immunoblot detection and quantification of BTK and GAPDH expression levels after siRNA and C481S transfection	α-BTK (±77 kDa) 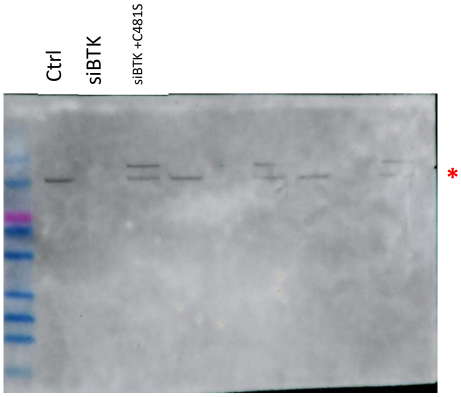	α-BTK (±77 kDa) 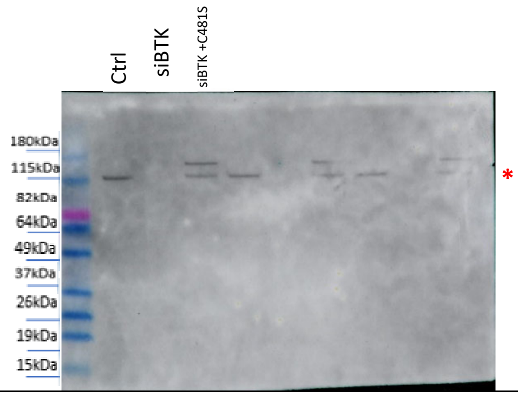

	α-GAPDH (±37 kDa) 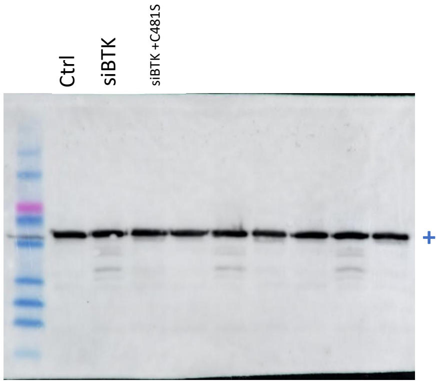	α-GAPDH (±37 kDa) 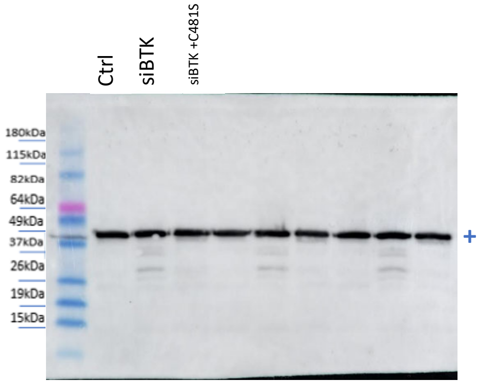
